# Carbon Fiber Biocompatibility for Implants

**DOI:** 10.3390/fib4010001

**Published:** 2016-01-08

**Authors:** Richard Petersen

**Affiliations:** Departments of Biomaterials and Restorative Sciences, University of Alabama at Birmingham, Birmingham, AL 35294, USA; Tel.: +1-205-934-1067

**Keywords:** carbon fiber, conductivity, resistivity, biocompatible, implant

## Abstract

Carbon fibers have multiple potential advantages in developing high-strength biomaterials with a density close to bone for better stress transfer and electrical properties that enhance tissue formation. As a breakthrough example in biomaterials, a 1.5 mm diameter bisphenol-epoxy/carbon-fiber-reinforced composite rod was compared for two weeks in a rat tibia model with a similar 1.5 mm diameter titanium-6-4 alloy screw manufactured to retain bone implants. Results showed that carbon-fiber-reinforced composite stimulated osseointegration inside the tibia bone marrow measured as percent bone area (PBA) to a great extent when compared to the titanium-6-4 alloy at statistically significant levels. PBA increased significantly with the carbon-fiber composite over the titanium-6-4 alloy for distances from the implant surfaces of 0.1 mm at 77.7% vs. 19.3% (*p* < 10^−8^) and 0.8 mm at 41.6% *vs*. 19.5% (*p* < 10^−4^), respectively. The review focuses on carbon fiber properties that increased PBA for enhanced implant osseointegration. Carbon fibers acting as polymer coated electrically conducting micro-biocircuits appear to provide a biocompatible semi-antioxidant property to remove damaging electron free radicals from the surrounding implant surface. Further, carbon fibers by removing excess electrons produced from the cellular mitochondrial electron transport chain during periods of hypoxia perhaps stimulate bone cell recruitment by free-radical chemotactic influences. In addition, well-studied bioorganic cell actin carbon fiber growth would appear to interface in close contact with the carbon-fiber-reinforced composite implant. Resulting subsequent actin carbon fiber/implant carbon fiber contacts then could help in discharging the electron biological overloads through electrochemical gradients to lower negative charges and lower concentration.

## 1. Introduction

In addition to well-known structural mechanical properties [1–3], carbon fibers have certain biocompatible properties that have been recognized clinically [4–7] through animal research [8–10] and experimentally in the lab [11–15]. Carbon fiber is lightweight with a density of 1.6–2.2 g/cm^3^ [1–3,16] compared to the density of compact bone at 2.0 g/cm^3^ [17]. Carbon fibers with a bendable small diameter, high-strength, and high-modulus material [1–3] can be molded with adaptation into complex curved spaces for multiple variations in applied use. Carbon fiber is a generic term referring to a family of fiber created by the pyrolysis of organic precursor fibers like rayon, polyacrylonitrile (PAN), and pitch in an inert environment [1–3,16]. Carbon fiber has a graphitic structure with strong crystallite covalent bonds that are highly anisotropic for exceedingly large mechanical properties along the axis direction but with weak van der Waals forces between layers for minimum mechanical properties in the transverse or perpendicular direction [1–3,16]. Therefore, in order to create a high modulus carbon fiber the orientation of the graphitic crystal can be improved by different types of thermal and stretching treatments [1–3,16]. For example, PAN precursor carbon fibers have strengths from 5.65 GPa to 2.4 GPa and modulus from 436 GPa to 230 GPa [1–3]. Due to the low density for carbon fibers and high mechanical properties carbon fibers can have specific strengths and moduli much stiffer and stronger than steel [1,3], Table 1. Because of such high specific strength and modulus, carbon fibers are used in high-performance composites in a variety of applications demanding lightness and high mechanical properties particularly in the aerospace and aircraft industries [1–3,18]. Further, carbon fibers have complete elastic recovery after unloading for excellent fatigue resistance [2,16]. The inert nature of carbon fibers produces a material with excellent moisture and chemical resistance at room temperature, but oxidization starts at higher temperatures in a range from 350–450 °C that increases with fiber impurities [1,2,16]. Due to the inert nature of carbon fibers, finishes similar to the polymer matrix of the reinforced composite are applied to form a thin 100 nm coating for improved wetting and impregnation of the carbon fiber [16].

Carbon fibers are good electrical conductors with electrical resistivities ranging from 9.5 × 10^−6^ Ωm to 18 × 10^−6^ Ωm [1–3]. Increases in purity of carbon fibers increase both electrical and thermal conductivity in addition to increased modulus [16]. Typical carbon fiber composites with an electrically insulating polymer matrix provide controlled electrical conductivity that is highest parallel to the fiber direction [3]. Because of the insulating polymer, perpendicular transverse conduction in a carbon fiber composite is low but still occurs due to a small fraction of contacts between fibers [18]. Electromagnetic interference (EMI) based on static electronic noise caused by changing voltages [2] can be prevented by using the conductive carbon fibers [2,18–20]. Copper with a resistivity of 1.7 × 10^−8^ Ωm [1] has much better conductivity than carbon fiber for PET/MRI radio frequency (RF) shielding but is responsible for reduced MRI image quality due to induced distortions by eddy currents generated [21]. On the other hand, carbon fiber with less conductivity for low frequencies reduces MRI gradient induced eddy currents, but still shows good RF shielding for higher frequencies [21]. In addition to excellent mechanical properties, carbon fiber is “gamma transparent” due to low atomic number that aids in development of MRI housings [21]. Further, carbon fiber composites can be studied by resistance measurements to sense damage for delamination and level of fiber breakage as electrical conduction is reduced [18,22]. Of particular interest is the in-plane resistivity of a carbon-fiber-reinforced composite at 5 Ωm [20] that is similar to bone longitudinal resistivity of 45 Ωm [23] when compared to titanium or titanium alloys at 4.2–19.9 × 10^−7^ Ωm [1] for potential improvements by electron transport design in biomaterial osseointegration with bone.

Carbon fibers have been studied biologically and used clinically in a wide variety of applications. By electrical conductivity, carbon fibers have been used for voltammetric recognition of biological molecules [24]. Also, a carbon fiber electrode has been used for neural recording [10]. Further, carbon nanofibers can provide electrical conduction for stimulation of cardiomyocytes [25] and carbon nanofibers independently improve the proliferation of cardiomyocytes [26]. Also, carbon fibers could be shown to enhance the wound healing process in both soft and hard tissues [8]. In the 1980s carbon fibers were used clinically as a scaffolding tool to induce tissue proliferation for tendon or ligament repair [27,28]. In fact, carbon fibers were tested with apparent biocompatible success for ligament replacements in human knee reconstruction demonstrating concentric fibrous layers surrounding a carbon fiber core of mechanically sound intact fibers [28]. However, carbon fibers' low transverse shear strength resulted in the formation of permanent debris fragments [27]. Consequently, carbon fibers were not accepted by the Food and Drug Administration for anterior cruciate ligament replacement [29]. Discontinuous chopped carbon fibers have previously been added into acrylic bone cement for mechanical testing with improvements in tensile strength, modulus, flexural strength, shear strength, fatigue strength, and impact toughness [11,12,15]. Further, carbon fiber-reinforced composite has been used for bone fracture repair by internal fixation with plates that have been shown to stimulate healing better than metal by allowing stress to be applied more uniformly as a lower modulus material [4,5,7].

## 2. Carbon-Fiber Composite Bone Implant Material

### 2.1. Materials and in Vivo Animal Model

Because of the potential advantages of developing high-strength biomaterials with a density closer to bone for better stress transfer and electrical properties that enhance tissue formation, an in vivo animal rat tibia implant test model was used to demonstrate possible biocompatible improvements for carbon fiber in reinforced polymer matrix composite material. Further, titanium-6-4 alloy implant as a standard clinical material was compared to better explain experimental differences. Epoxy/carbon-fiber unidirectional composite 1.5 mm diameter rods were placed for two weeks using a rat tibia test design previously investigated [30]. As a result, past tissue slides for titanium-6-4 alloy (90% titanium; 6% aluminum; 4% vanadium) 1.5 mm diameter implant screws (Walter Lorenz Surgical Inc., Jacksonville, FL, USA) were available to compare changes [30] and quantify percent bone area (PBA) at a specific intramedullary distance from the implant using Bioquant software (Nashville, TN, USA). The unidirectional carbon-fiber-reinforced composite was manufactured with 60 volume percent fibers and 40 volume percent bisphenol epoxy (Aerospace Composite Products, Livermore, CA, USA).

### 2.2. Animal Testing

Ten male Sprague-Dawley rats weighing 350 g to 375 g were obtained for each group at different times to make PBA measurement by first testing the titanium alloy controls and then subsequent bisphenol-epoxy/carbon-fiber-reinforced composite rods. Two additional rats were investigated for separate histology imaging views of the epoxy/carbon-fiber composite. The animals were anesthetized and also given intraperitoneal anesthetics. A fine incision was placed on the medial-proximal surface of the tibia above the tibial protuberance so that tissue could be turned back to reveal the flat tibia surface underneath the joint. A slow-speed surgical handpiece with a No. 4 round bur and warm saline were operated for a small hole drilled into the tibia 8 mm proximal to the tibial cortical bone protuberance. A 1.3 mm diameter surgical implant twist drill bit was then used. The size of the hole in the medial aspect of the tibia was enlarged with a No. 6 round bur. The titanium-6Al-4V screws were rotationally placed by twisting into the opposite cortical bone. The epoxy-polymer/carbon-fiber-reinforced composite rods were cut to 5.0 mm lengths, cleaned with ultrasonic equipment and autoclaved for sterilization. Ultrasonic cleaning was not employed for two separate tibia tests that were sectioned horizontally. A 1.5 mm surgical implant twist drill bit was used to make a hole through the medullary canal and the opposite cortical shaft similarly to the titanium implants. The bisphenol epoxy/carbon-fiber-reinforced composite rods were placed by hand pressure and then tapped securely. The muscle layers were closed with resorbable sutures and the skin with surgical staples.

### 2.3. Histomorphic Analysis

After 14 days, rats were euthanized, tibiae were detached, cleaned of soft tissue, and imaged by photographs. Subsequent tibial specimens were fixed in phosphate-buffered paraformaldehyde for at least 12 h. Specimens were then dehydrated with progressive alcohols under vacuum, cleaned with xylene, infiltrated, and embedded with methylmethacrylate and polymerized by ultraviolet light. Samples were prepared by cutting and grinding that gave a lateral section of the implant. Final sample thickness was less than 60 μm mounted on clear plastic slides. Slides were stained with toluidine blue that identifies live bone. Further, Sanderson's stain was applied on two extra rat tibia slides not part of the statistical analysis and cut horizontal at right angles through the composite implants. Percent Bone Area (PBA) was measured as the area of bone within 0.8 mm and 0.1 mm of the implant inside the bone-marrow space of the tibia and between the cortical bone plates. The distance of 0.8 mm was determined as an approximation of the physiologic tibial cortical-plate thickness for the Sprague-Dawley rats in the experimentation. The distance of 0.1 mm was considered as a physiologic estimate for initial osseointegration with the implant and measure of oxygen diffusion through osseointegrating bone. BioQuant Software (Nashville, TN, USA) measured the data from the slides.

### 2.4. Statistics

**Figure 1:**
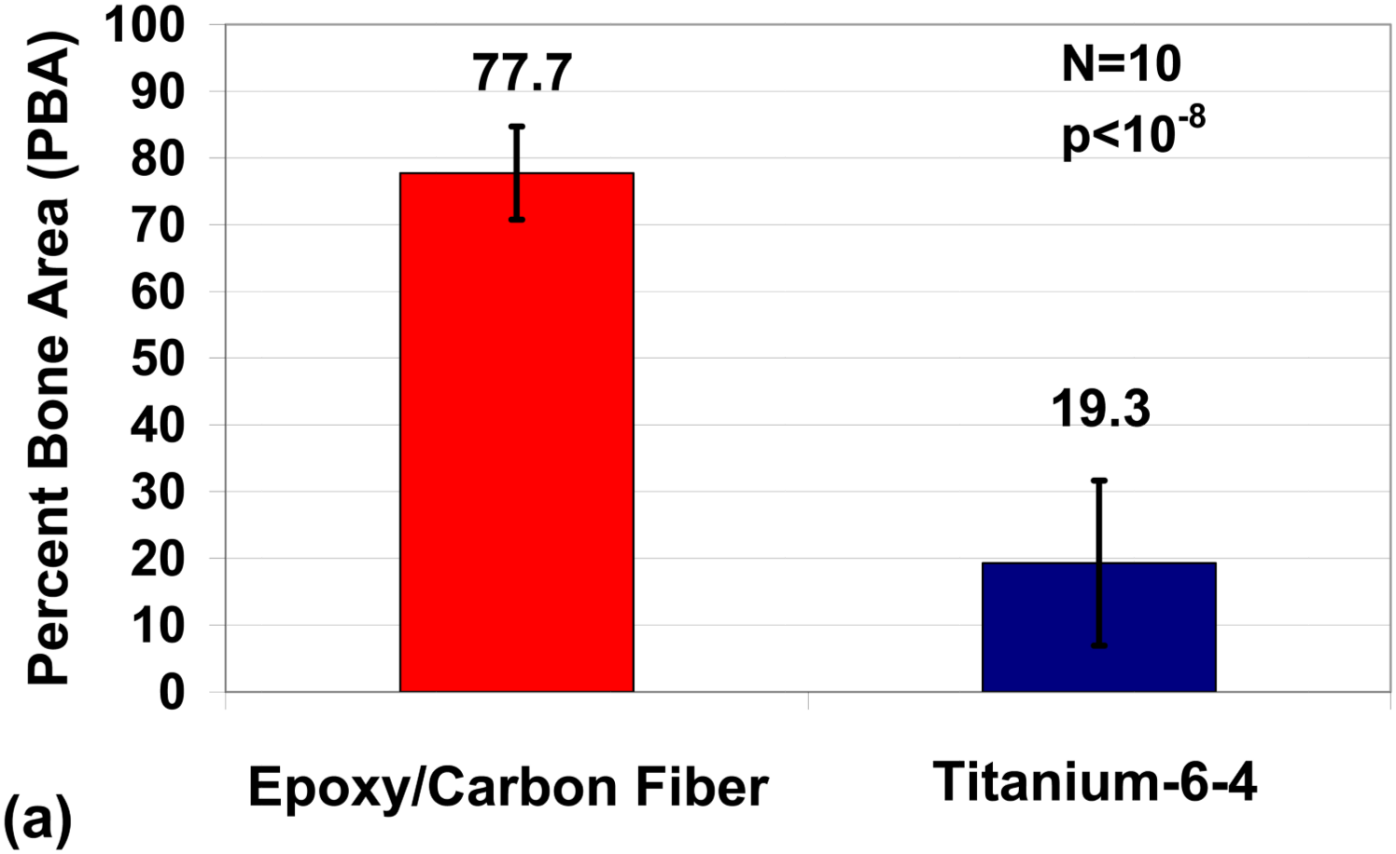

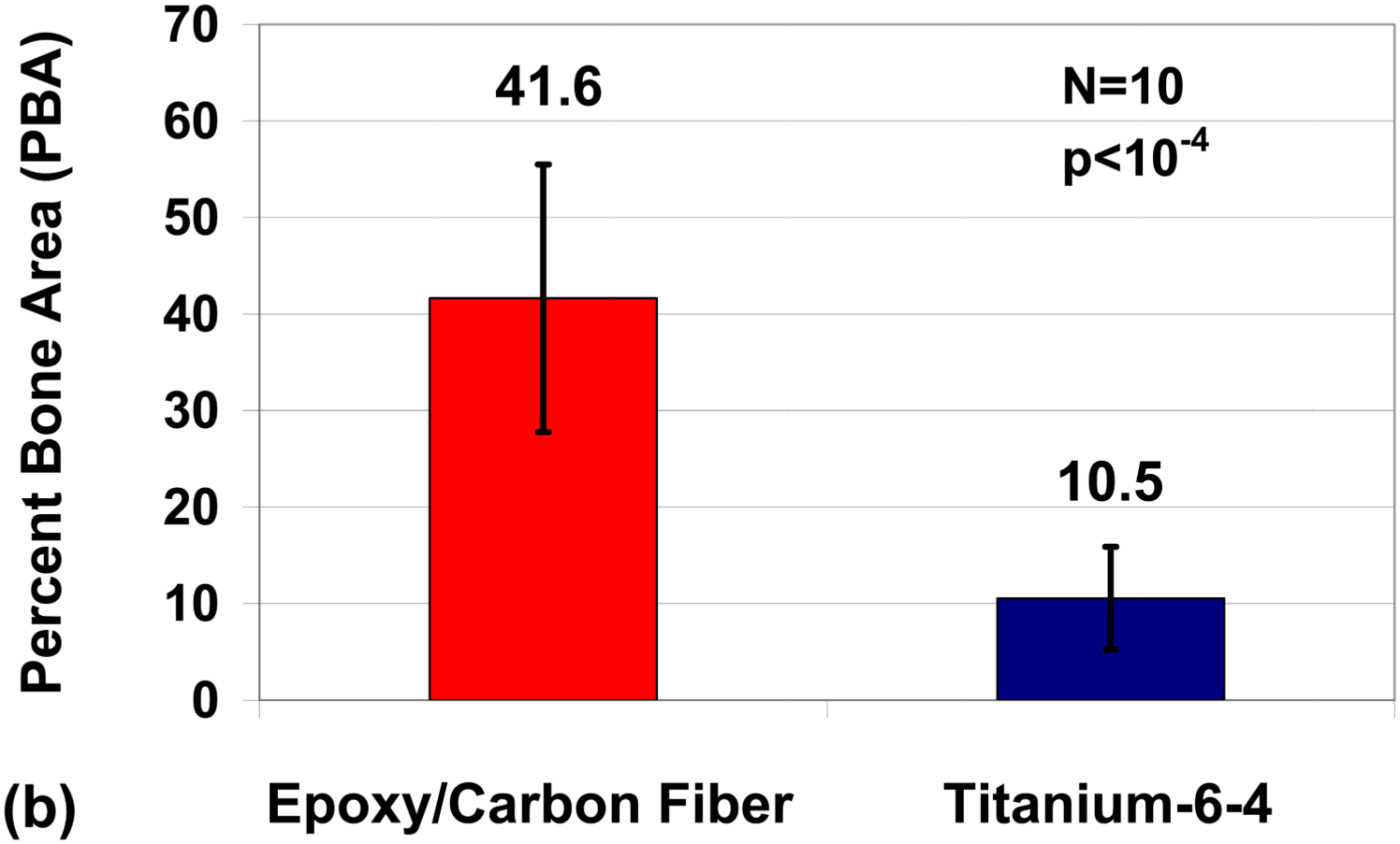
Implant PBAs comparing epoxy/carbon-fiber-reinforced composite to Ti-6Al-4V alloy (**a**) Distance 0.1 mm from implant; (**b**) Distance 0.8 mm from implant.

Differences between groups were calculated using a t-test with unequal variances with the marginal level of uncertainty set at α = 0.05. Significant statistical differences were found for tibia PBA results between epoxy/carbon-fiber-reinforced composites compared to titianium-6-4 alloy, Figure 1a,b. At a distance of 0.1 mm from the implant, PBA increased from 19.3 ± 12.3 to 77.7 ± 7.0 when comparing titanium alloy to the carbon-fiber-reinforced composite *p* < 10^−8^. At distance of 0.8 mm from the implant, PBA increased from 10.5 ± 5.3 to 41.6 ± 13.9 when comparing the titanium alloy to the carbon-fiber-reinforced composite, *p* < 10^−4^. The epoxy/carbon-fiber-reinforced composite and titianium-6-4 alloy both increased PBA almost double from 41.6 to 77.7 and 10.5 to 19.5 respectively when comparing the implant distance of 0.8 mm to the distance of 0.1 mm.

### 2.5. Imaging

**Figure 2:**
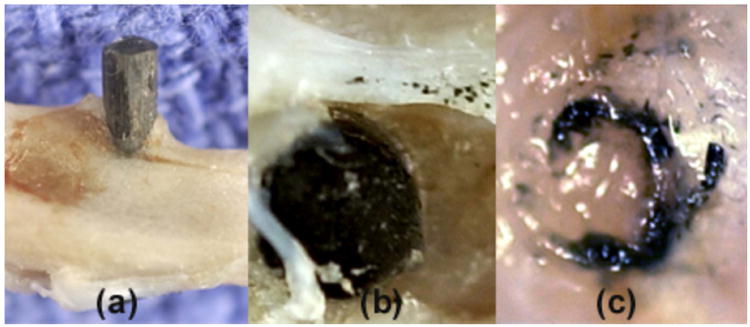
Photographs (**a**) epoxy/carbon-fiber-reinforced composite extends above tibial cortical bone with bone enhanced to grow upward on the side of the exposed carbon-fiber implant; (**b**) implant extending above cortical bone shows excess osteoid production apparently encouraged from small carbon fiber fragments; (**c**) soft tissue covering the cortical bone formed a toughened seal on the end of the implant associated with the carbon fiber fragments.

**Figure 3:**
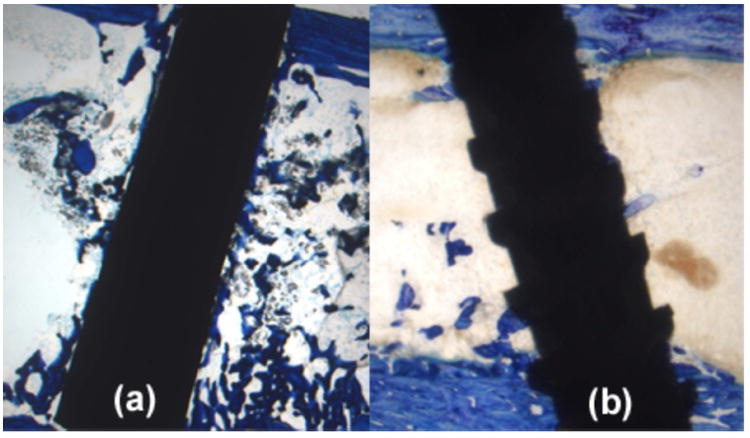
Lateral toluidine blue stain section 2× magnification rat tibia bone marrow and implant (**a**) Typical epoxy/carbon-fiber-reinforced composite; (**b**) Typical titanium-6Al-4V alloy.

**Figure 4:**
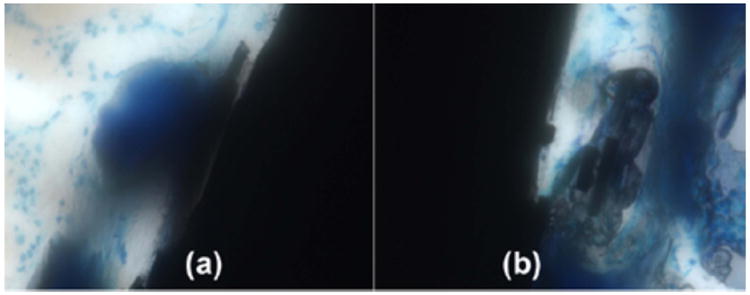
Lateral histology section at 40× magnification by toluidine blue stain for epoxy/carbon-fiber-reinforced composite implant with carbon fibers cleaved and pulled perpendicularly away from the implant. (**a**) Carbon fibers are cleaved transverse to the long direction of the unidirectional composite implant; (**b**) Bone osseointegrates completely around small carbon fiber fragments.

**Figure 5:**
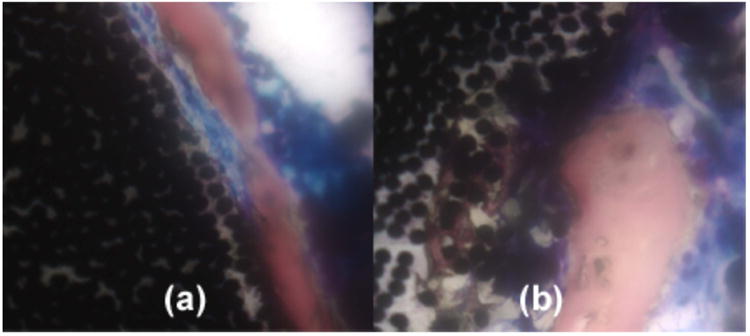
Horizontal histology section with Sanderson's stain at 40× magnification. (**a**) Bone osseointegration at the implant surface can degrade the polymer matrix and pull carbon fibers outward; (**b**) Bone has osseointegrated at an implant surface defect to degrade and replace polymer matrix with osteoid that has even surrounded individual carbon fibers.

Imaging characterization was performed by photography in Figure 2a–c and from histological slides in Figures 3–5. Imaging highlighted biocompatibility possibilities with significant osseoconductive reactions for the epoxy/carbon-fiber-reinforced implants that surpassed the titanium-6-4 alloy commercial bone implant screws. Bone growth was encouraged along the lengths of the entire epoxy/carbon-fiber-reinforced implant surfaces and grew above cortical bone surface levels on the implant and in part over the ends of many exposed carbon-fiber-reinforced rods, through the tibia bone-marrow space, and filled in drilling space between the implant and cortical bone. Photograph imaging demonstrates calcifying osteoid in Figure 2a,b that would follow the carbon-fiber-reinforced composite implant surface above the upper cortical bone plate and sometimes partially grow over the implant end. Separate tests not included in the statistical analysis retained small amounts of epoxy/carbon-fiber fragments along the implant before surgery which resulted in an exuberant osteoid reaction on the cortical plate over the implant end in Figure 2b. A simple dissection around the entire tough fibrous soft tissue that covered the end of an epoxy/carbon-fiber-reinforced implant showed that soft tissue integration is related to carbon-fiber fragments in the photograph for Figure 2c.

For histology evaluation at 2× magnifications the epoxy/carbon-fiber-reinforced implant, Figure 3a, demonstrates extensive osseointegrating bone formation along the total implant surface. Conversely, the titanium alloy, Figure 3b, shows simply small fragments of bone integrating along the implant surface.

The epoxy/carbon fiber implants at 40× magnifications, Figure 4a,b, showed transverse fiber fracture with fiber fragments. However, all carbon-fiber fragments exhibited stimulated bone growth at the fiber surface. Some cleaved carbon fiber fragments were even surrounded by growing bone.

Low oxygen tissue concentrations create acids during mitochondrial energy synthesis so that epoxy polymer of the composite is degraded and pulled away from the implant surface by attached bone, Figure 5a. Carbon fiber can also degrade transversely into a fine particulate smear layer line on the outside surface of the implant immediately next to the bone. Further, epoxy polymer is degraded so that noncalcified osteoid can grow into the implant and even surround individual carbon fibers for enhanced osseointegration, Figure 5b.

## 3. Biological Implant Considerations

### 3.1. Metabolic Cell Oxygen Demands

Highly significant PBA increases by the carbon-fiber-reinforced composite compared to the titanium alloy implant can be evaluated relative to contributions from the carbon fibers. Further, bone and osteoid extensively enhanced by contact with carbon fibers and highly improved soft tissue sealing response warrant explanation regarding stimulating tissue growth. Pertaining to carbon fiber electrical conductivity, cell metabolism with low oxygen concentrations for production of electrons is evident at an implant surface. For example, as a uniform gauge capillary distance is a measure of lower oxygen concentration and increased acid or lower pH such that zero O_2_ concentrations develop at about a 0.2 mm tissue distance [31–33]. Oxygen concentrations become lower as the distance increases from the blood supply creating intracellular metabolic production of electrons and acid by the cellular organelle called mitochondria [31–35]. The lower oxygen concentrations near the implant surface are unable to satisfy intracellular mitochondrial metabolism demands during the formation of electrons and protons through adensosine triphosphate (ATP) energy synthesis to form water [34,35], Equations (1) and (2).

(1) $$O_{2}+2e^{-}+2H^{+}=H_{2}O_{2}$$

(2) $$H_{2}O_{2}+2e^{-}+2H^{+}=2H_{2}O$$

### 3.2. Cell Motility

**Figure 6:**
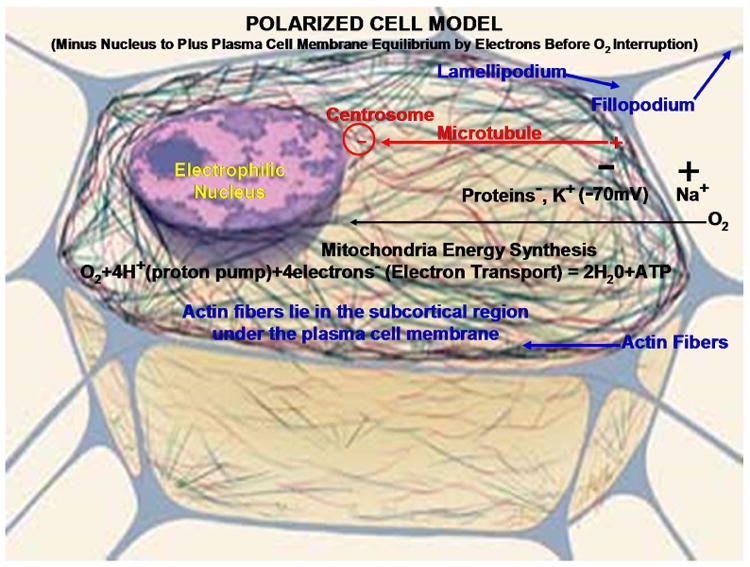
Polarized mitochondrial energy synthesis cell model with cytoskeleton fibers and lamellipodium projections and fillopodium focal adhesions. With permission from National Institutes of Health/Department of Health and Human Services. All subsequent text labels and arrows created by author.

Cellular motility can be directionally controlled by chemical gradients as chemotaxis [36] while proteins can contract to create cell movement [37] that can both be associated with electrons created by the energy producing mitochondria. Consistent with valence bond theory, a covalent bond forms when two atoms come closer together so that electrons pair in overlapping orbitals and are attracted to both atomic nuclei [38,39]. Free radicals are molecules with an unpaired electron that form from reactive oxygen species including H_2_O_2_ and have demonstrated ability as chemotactic factors which bind with cell membranes by polymerization and contraction of protein actin organic-carbon fibers for polarized cell movement toward H_2_O_2_ and other reactive oxygen species [40–42]. In addition, the motile cell is polarized by microtubule protein organic-carbon fibers extending from the centrosome near the nucleus to the peripheral cell membrane edges [43–45], Figure 6.

The polarization extends with protein actin fiber projections for adhesions between the extracellular matrix that contract together and pull in the forward direction [43–46]. The cell extensions are long lengthened lamellipodia and short adhesive fillopodia made from actin fibers that polymerize at the advancing edge to pull the cell forward [43,44,46]. As a part of cellular physiology, the outer plasma cell membrane develops a voltage potential with a negative charge on the inside and positive charge on the outside of the cell [47]. Cytoskeleton protein microtubule fibers and actin protein fibers are polarized positively near the outer plasma cell membrane to lengthen [44–46] and negatively by microtubules toward the organizing centrosome near the nucleus [45,48]. A clear long-range static electric field is created on the mitochondria and also on the microtubules that arise in close contact [49] developing a possible delocalization channeling mechanism for the electron transport chain during periods of mitochondrial oxidative stress when an excess of electrons build up. Again, polymerization of actin fibers at the positive end with the lamellipodia protrusions and small focal fillopodia that form adhesions with the extracellular matrix result in molecular contractions during bonding to provide forward movement [43,44,46]. Conversely, as depolymerization occurs at the negative ends of the actin fibers on the rear edge of the cell movement, small adhesions break free making actin monomers available to be recycled for polymerization at the forward positive actin extensions [44,46].

## 4. Biomaterial Implant Considerations

### 4.1. Carbon Fiber Biocompatible Conductivity vs. Metal Acids

Because carbon fibers are electrically conductive [1–3] an insulating epoxy polymer coating then develops micro-circuits in a polymer matrix composite [3,18]. Subsequent excess mitochondrial electrons during low oxygen concentrations are possibly able to move and stream fast through carbon fibers electrochemically to areas of lower negative charge and lower electron concentrations. Bone cells could then have a tendency to move toward carbon fibers and release excess electrons created from the electron transport chain during mitochondrial energy synthesis concurrent with low oxygen concentration to prevent production of damaging free radicals. As electrons are released from the cells under respiratory stress into carbon fibers, free-radical chemotactic influences would have a tendency to move cells in the same direction as actin filaments grow by polymerization outward from the cell toward the implant. Further, conductivity provides an opportunity for removing inflammatory surgical free radicals to form possible covalent bonds with other exposed unpaired electrons [50]. Most obviously, carbon fibers apparently act as a permanent semi-antioxidant to redistribute electrons and free radicals that could interfere with bone growth.

According to Equations (1) and (2) hydrogen ions or hydronium ion in water can possibly form when oxygen is deficient during energy metabolism. However, with the metal implant hydrogen ions should be produced at a greater rate than with a polymer matrix composite due to the formation of metal cations (M^+^) and electrons (e^−^), Equation (3) [51–53] that dissolve into a biologic fluid. Aqueous concentrations of residual dissolved molecular oxygen in the tissue react and remove electrons to form hydroxyl anions [51–53], Equation (4) that helps drive corrosion through Equation (3) [53] and lower oxygen concentration even more. Further, metal cations are removed to polarize water forming a Lewis acid, Equation (5) [53–55] that can then accelerate corrosion through Equation (3).

(3) $$M=M^{+}+e^{-}$$

(4) $$O_{2}+2H_{2}O+4e^{-}=4{\text{OH}}^{-}$$

(5) $$M^{+}+H_{2}O=(M^{+})({\text{HO}}^{-}-H^{+})=M^{+}({\text{OH}}^{-})+H^{+}$$

For the polymer matrix unidirectional carbon-fiber-reinforced implant increasing acid with low pH in the microenvironment next to the carbon fiber can then create breakdown conditions of the generally chemically resistant passive carbon fiber with weak transverse strength to initiate fiber fracture. Figures showing bone to implant attachments indicate that covalent bonding with the carbon fibers by electron pair sharing is a possibility while polymer covalent bonding also appears feasible. Further, mechanical retention occurs as polymer degrades for achievable strong bone ingrowth around individual carbon fibers. On the other hand, titanium electron bonding is ionic with mineralization between bone and the TiO_2_ surface oxide layer.

### 4.2. Polymer Estrogen Influence

Carbon fiber-reinforced composites provide an additional benefit for design application with the polymer matrix. Estrogen factors are present from bisphenol polymers [56–61] with a backbone derived from one of the first synthetic estrogens [56]. Subsequent physiologic actions of estrogen on bone include skeletal growth, increased osteoblast activity, and retained Ca^2+^ and HPO_4_^2−^ mineralization due to organic bone matrix formation [62]. Further, estrogen and a precursor for resin, bisphenol A, protects against ovary degeneration, uterine shrinking, and bone loss in a concentration dependent manner [60,62]. Bisphenol A has been shown to increase adult rat femur length without loss of strength [63]. For a biologic comparison, the outer plasma cell membrane is composed of lipids, proteins, and carbohydrates [62] all of which are similar in nature with molecular polarity closer to the bisphenol epoxy than a metal. Also, cholesterol is a precursor to estrogen and found in the membrane to help maintain membrane fluidity [62]. Closed shell molecules attract one another through van der Waals forces because of the partial charges in polar covalent chemistry that further includes the small nonpolarity electronegative differences in hydrocarbons through multipolar effects [64] resulting in related molecular chains attracting one another. Subsequent similarities in molecular forces of attraction then exists in variation between the thermoset cure bisphenol polymers with the plasma cell membrane [62] and organic portions of the bone matrix as forms of material biological function [9]. Consequently, bone-marrow precursor cells for the bone-forming osteoblasts would apparently be recruited toward the bisphenol epoxy implant composite by similar chemical molecular structures to then help form mature bone [9].

### 4.3. Fiber-Reinforced Composite Design Capability

According to a well-known biologic response termed “Wolff's law” bone remodels in reaction to mechanical loading so that the newly formed bone is better modified to subsequent applied forces [65]. Metal has a much higher stiffness than cortical bone so that stresses are not transferred uniformly [65]. Subsequent loading is thus carried to a far greater extent through a fixed rigid metal bone plate rather than by nearby bone [65]. The modulus for a metal bone plate is between 110 GPa and 220 GPa compared to human long cortical bone of around 17–20 GPa [66]. Cellular bone formation and bone loss are balanced so that when higher loads are applied osteogenic bone formation occurs to counteract the extra force [65–67]. Consequently, with metal plates, the bone fracture is “shielded” or under-stressed and prevented from healing normally even for tissues ingrown into the fracture site by resorbing into weaker bone according to Wolff's law [65–67].

Although preliminary clinical testing in the early 1990s to heal fractures with epoxy/carbon-fiber-reinforced composite bone plates demonstrated unique biocompatibility when compared to titanium or stainless steel [4,5], development proceeded slowly. However, recently a new interest in carbon-fiber-reinforced composites has emerged to reduce stress shielding common with metal bone plates [7,66,67]. Further, polymer/carbon-fiber-reinforced bone plates are radiolucent to provide X-ray density sufficiently low for relatively easy visualization of bone callus formation in the fracture area that is not possible with dense, radio-opaque metal plates [7,66]. With X-ray radiolucent carbon-fiber-reinforced bone plates the callus can be evaluated more closely to allow better clinical judgment for patient care compared with metal plates [7]. Also, machining is accomplished much easier with a carbon-fiber-reinforced composite material than metal so that screws can be designed at angles with multiple directions to improve coupling between the plate and bone, which is particularly difficult at the distal bone ends [7]. Cold welding is another problem with metal bone plates and metal screws that cannot occur with a carbon-fiber-reinforced bone plate [7].

Designing with fiber-reinforced composites becomes an important factor when considering the different applications and widespread needs for various medical devices that range from temporary fracture bone plates that need eventual removal to long-term fixation with osseointegration by bone implants or bone cement grouting material. Modulus, strength, and fracture toughness can be modified according to fiber volume percent, fiber directions and different types of mechanical properties for the fibers employed [68–70]. Further, fiber length can be used to determine material composite strength, modulus, and fracture toughness [71]. Of importance, fiber-reinforced composite resistivity/conductivity can similarly even be included in the designing phase [1–3]. The properties for resistivity or conductivity are particularly evident with carbon fibers through insulating polymer matrix biocircuits to account for excess electron tissue accumulation that needs redistribution for proper medical device healing depending on removal time or need for long-term fixation.

### 4.4. Carbon Fiber Percolation Threshold with Cell Motility

For a carbon-fiber-reinforced composite, the polymer is insulating and the carbon fibers are conductive. With a direct current source the insulation-conduction transition is described by a critical concentration of carbon fibers where conductivity suddenly increases at the percolation threshold [72]. Contacts between carbon fibers provide conduction higher along the long axis compared to the transverse directions [73]. As the carbon fiber volume percentage decreases, the resistivity increases in all of the directions [73]. Longitudinal resistivity for an epoxy matrix unidirectional 60 volume percent carbon-fiber-reinforced composite similar to the implant animal tibia study has been measured between 7.5 Ωm and 10.0 Ωm [73]. Further, tunneling between carbon fiber contacts creates conductivity before actual fiber contacts are made [73] that would appear to be the case in a cell actin biocarbon-fiber system with a carbon fiber biomaterial. However, as a practical consideration the chemotactic influence of cell movement by electrons toward carbon fibers and combined actin fiber polymerization cell movement toward making biomaterial carbon fiber contacts precludes a valid percolation threshold at the cell/biomaterial interface. Consequently, cell electron conduction is achieved even with minimal carbon fiber fragments by actin biocarbon fibers at extremely apparent low electron concentrations thereby equalizing electrons to areas of lower concentrations and lower negative charges. Regardless of biomaterial well-known carbon-fiber percolation threshold, even minimum biomaterial conductivity is osteogenic by removing highly damaging excess biological electrons.

## 5. Conclusions

Carbon fiber reinforcement as an electrically conductive microcircuit in a polymer matrix composite has shown experimental reliability to stimulate tissue growth by removing excess electrons produced under respiratory stress. Most precisely, oxygen is the ultimate electron acceptor and required during efficient energy synthesis, otherwise free radicals and acid result that can be damaging to cells. Subsequent carbon fiber conductivity then has possible biocompatible properties in removing excess damaging electrons through electrochemical gradients to areas of lower negative charges and lower concentrations. Further, carbon fiber has the ability to osseointegrate with live bone.

## Figures and Tables

**Table 1:** Specific Properties for Carbon Fiber and High-Strength Steel.

Material	Specific Gravity (g/cm^3^)	Tensile Strength (GPa)	Specific Strength (GPa)	Modulus of Elasticity (GPa)	Specific Modulus (GPa)
Carbon Fiber	1.6–2.2	1.5–5.65	0.70–3.12	228–790	106–407
Steel Wire	7.9	2.39	0.30	210	26.6
